# In children and adolescents with temporomandibular disorder assembled with juvenile idiopathic arthritis - no association were found between pain and TMJ deformities using CBCT

**DOI:** 10.1186/s12903-021-01870-z

**Published:** 2021-10-12

**Authors:** J. Fischer, T. A. Augdal, O. Angenete, E. G. Gil, M. S. Skeie, A. N. Åstrøm, K. Tylleskär, K. Rosendahl, X.-Q. Shi, A. Rosén, The NorJIA (Norwegian JIA Study — Imaging, oral health, and quality of life in children with juvenile idiopathic arthritis).

**Affiliations:** 1 Department of Clinical Dentistry, The Faculty of Medicine, University of Bergen, Årstadveien 19, 5009 Bergen, Norway; 2 Department of Radiology, University Hospital of North Norway, Tromsø, Norway; 3 Department of Radiology and Nuclear Medicine, St. Olav Hospital HF, Trondheim, Norway; 4 Department of Circulation and Medical Imaging, Faculty of Medicine, Norwegian University of Science and Technology, Trondheim, Norway; 5 Center for Oral Health Services and Research, Mid-Norway (TkMidt), Trondheim, Norway; 6 Public Dental Service Competence Centre of Western-Norway (TkVest), Bergen, Norway; 7 Paediatric Clinic at Haukeland University Hospital, Bergen, Norway; 8 UiT Arctic University of North Norway, Tromsø, Norway; 9 Department of Oral and Maxillofacial Radiology, Faculty of Odontology, University of Malmö, Malmö, Sweden; 10 Department of Oral and Maxillofacial Surgery, Haukeland University Hospital, Bergen, Norway

**Keywords:** Juvenile idiopathic arthritis, Temporomandibular joint arthritis, Temporomandibular disorder, Temporomandibular joint deformity, Cone-beam computed tomography

## Abstract

**Background:**

Children and adolescents with juvenile idiopathic arthritis (JIA) may suffer from temporomandibular disorder (TMD). Due to this, imaging diagnosis is crucial in JIA with non-symptomatic TM joint (TMJ) involvement. The aim of the study was to examine the association between clinical TMD signs/symptoms and cone-beam computed tomography (CBCT) findings of TMJ structural deformities in children and adolescents with JIA.

**Methods:**

This cross-sectional study is part of a longitudinal prospective multi-centre study performed from 2015–2020, including 228 children and adolescents aged 4–16 years diagnosed with JIA, according to the International League of Associations for Rheumatology (ILAR). For this sub-study, we included the Bergen cohort of 72 patients (32 female, median age 13.1 years, median duration of JIA 4.5 years). Clinical TMD signs/symptoms were registered as pain on palpation, pain on jaw movement, and combined pain of those two. The severity of TMJ deformity was classified as sound (no deformity), mild, or moderate/severe according to the radiographic findings of CBCT.

**Results:**

Of 72 patients, 21 (29.2%) had pain on palpation at and around the lateral pole, while 41 (56.9%) had TMJ pain upon jaw movement and 26 (36.1%) had pain from both. Of 141 TMJs, 18.4% had mild and 14.2% had moderate/severe structural deformities visible on CBCT. CBCT findings were not significantly associated with either the pain on palpation or the pain on jaw movement. A significant difference was found between structural deformities in CBCT and the combined pain outcome (pain at both palpation and movement) for both TMJs for the persistent oligoarticular subtype (p = 0.031).

**Conclusions:**

There was no association between painful TMD and CBCT imaging features of the TMJ in patients with JIA, but the oligoarticular subtype of JIA, there was a significant difference associated with TMJ pain and structural CBCT deformities.

**Supplementary Information:**

The online version contains supplementary material available at 10.1186/s12903-021-01870-z.

## Background

Juvenile idiopathic Arthritis (JIA) is a heterogeneous condition that includes all forms of chronic arthritis of unknown origin with a duration of more than six weeks and an onset before 16 years of age [1, 2]. The reported prevalence is around 1–2 per 1000 children with girls more frequently affected than boys [3–5], and the condition is characterized by chronic synovial inflammation, with potential risk of developing progressive joint destruction and serious functional disability [1, 6, 7]. JIA includes seven subtypes (systematic arthritis, oligoarthritis (persistent or extended), rheumatoid factor negative polyarthritis, rheumatoid factor positive polyarthritis, psoriatic arthritis, and enthesitis-related arthritis) with different (though overlapping) characteristics. The estimated prevalence of temporomandibular joint (TMJ) arthritis in children and adolescents with JIA varies widely between 17 and 92%, of which a high proportion of cases appear to be clinically silent [8, 9]. TMJ arthritis is often combined with temporomandibular disorder (TMD), which is defined as muscular tensions from the surrounding muscles, or inflammation and/or destructive deformities in the TMJs of these patients, or a combination of the two [10]. Children and adolescents with JIA are more likely to suffer from TMD than their healthy peers, which means that children and adolescents with JIA are more likely to have impaired oral health [11–14]. In a recently published article from our multi-centre study, we found that 40% of patients with JIA aged 6–16 years old experienced TMD [15]. An even higher TMD figure of 83% was reported in a cohort of Brazilian adolescents with JIA [16], while a Danish study revealed that 38–53% of patients with JIA (median age 6.6 years) experienced orofacial symptoms and dysfunction due to TMJ arthritis and/or muscular tensions [17]. Cone-beam computed tomography (CBCT) has been used as a 3D diagnostic modality for nearly two decades [18, 19] and the radiation doses are of this method are, in general, lower than that of conventional CT. For TMJ screening, CBCT imaging has been reported to require a 30% lower dosage and give a better image quality than CT [20]. In a retrospective study by Cho and colleagues including 282 children and adolescents aged 10 – 18 years, the authors found an association between TMJ condylar deformities and TMJ symptoms or reduced mouth opening capacity [21]. Another CBCT-based study showed that children and adolescents (10–19 years) with TMD had more erosive cortical bone changes than same-aged pre-orthodontic controls with malocclusion [22], and the same study also highlighted that pre-orthodontic participant with malocclusion presented solid radiographic signs. Although CBCT is the method of choice for assessing TMJ deformity, examples of CBCT use in children and adolescents with JIA-associated TMD are sparse. JIA may result in TMJ deformity and affect mandible development as well as chewing function. Therefore, early diagnosis and treatment of TMJ deformity are of clinical importance. However, there are no diagnostic guidelines available on whether CBCT is indicated for JIA patients or for which group of patients it is indicated. Clinical symptoms may serve as predictors for justified CBCT examination.

Therefore, the aim of this study is to examine the association between clinical signs/symptoms of TMD and structural TMJ deformities found from CBCT in this patient group.

## Methods

We followed the strengthening the reporting of observational studies in epidemiology (STROBE) reporting guideline. This cross-sectional study is part of a longitudinal prospective multi-centre study performed from 2015–2020, including 228 children and adolescents aged 4–16 years, diagnosed with JIA according to the International League of Associations for Rheumatology (ILAR) [1]. Excluded from the study were those with congenital facial anomalies and/or major medical co-morbidities and those who did not consent to participate. The unselected material was retrieved from the Bergen NorJIA cohort of children and adolescents with JIA (n = 72) from 2015–2017 and included standardized assessments of TMD as part of a broader oral health examination.

Clinical TMD examinations were performed by using a shortened version of the Diagnostic Criteria for Temporomandibular Disorders (DC/TMD) Axis I [23] and the self-assessment questionnaire recommended by TMJaw for clinical TMJ assessment in patients diagnosed with JIA [24]. The reason for this combination of diagnostic tools is that the DC/TMD tool alone is reported to have weak validity for TMJ assessment. Therefore, this tool can also identify disc displacement (low sensitivity) and degenerative joint disease (low sensitivity and specificity). To avoid systemic error, reliability results from various calibration exercises in TMD diagnostic prior to and during the study period are described in our previous publication [15].

**Fig. 1:**
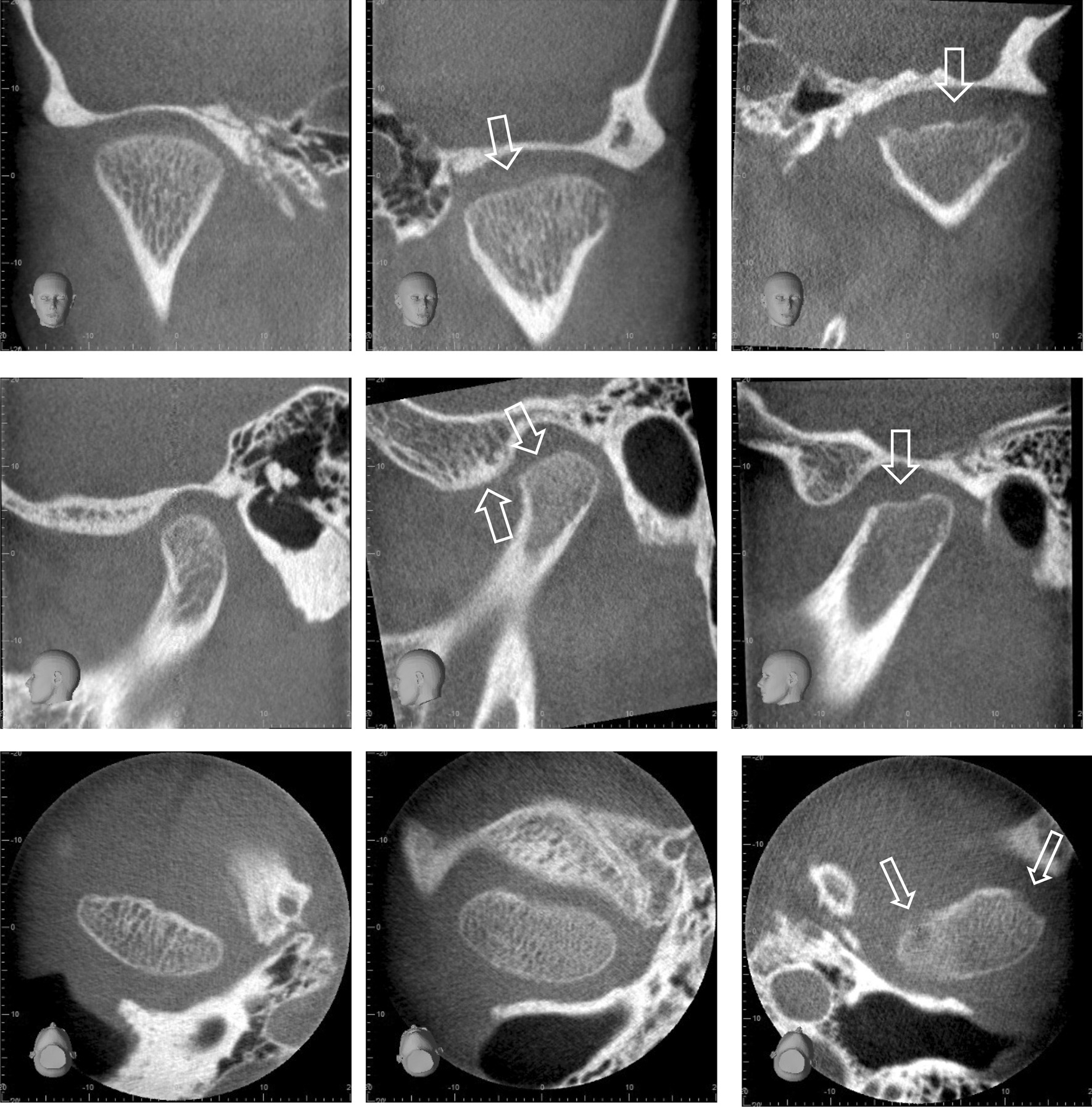
Examples of temporomandibular joint (TMJ) deformity on cone-beam computed tomography (CBCT)

During clinical examination, we determined whether there was pain on direct palpation at and around the lateral pole. Moreover, we asked the patient whether he/she currently experienced TMJ and masticatory muscle symptoms on vertical, lateral, and or protrusive jaw movements, and we also registered combined pain (pain on palpation and pain on jaw movement). The CBCT examination was performed using a 3D Accuitomo 170 (Morita) with a field of view of 4 × 4 cm and a voxel size of 80 µm. The exposure parameters were adjusted for each individual patient. All images were exported into iDixel One Volume Viewer (Version 2.6.0 Morita), and analysed by an experienced paediatric radiologist (TAA, 13 years of experience in paediatric imaging) with additional information in the image masked. The overall impression of TMJ deformity was categorized into one of three groups based on the radiographic appearance in the condyle and temporal parts: sound = normal anatomical variation, mild = slight flattening of the fossa/eminence or condyle, or minor joint surfaces irregularities, moderate/severe = apparent deformation of fossa/eminence or condyle, apparent reduction of condyle volume or more severe joint surface irregularities. Examples of typical cases are shown in Fig. 1.

### Statistical methods

Descriptive statistics were reported as mean with SDs, percentages, or median (ranges). For analyses, we dichotomized the TMJ deformity variable to absent or present due to low number of cases in the mild and moderate/severe groups. Associations between localized pain (TMD) and structural deformities visible in CBCT were examined using Fisher’s exact/chi-square test and an independent/two-sample t-test as appropriate. Statistical analyses were performed using SPSS version 25 (IBM Corporation, New York, NY, USA). All tests were two-sided and statistical significance was set at 5% (p ≤ 0.05).

### Ethical considerations

Ethical approval for this study was obtained from the Regional Committee For Medical And Health Ethics (REC west), Universitetet i Bergen, Det medisinske fakultet, Postboks 7804, 5020 Bergen, reference number: 2012/542/REC west. Written informed consent was obtained from the parents or legal representatives of the children and adolescents. The study was registered in ClinicalTrials.gov (No: NCT03904459).

## Results

A total of 72 children and adolescents (44% girls) and a median age of 13.1 years (range 5.9–16.5 years) were included (Table 1). The most prevalent ILAR categories were persistent oligoarthritis, present in 31 (43.7%) of the participants, and rheumatoid factor negative polyarthritis (RF-negative), present in 14 (18.3%) of the participants. None had rheumatoid factor positive polyarthritis. No statistically significant differences in the presence of TMD according to JIA category were observed (p = 0.837).

**Table 1:** Characteristics of participants with juvenile idiopathic arthritis (JIA) in relation to temporomandibular disorder (TMD)

	Bergen cohort n = 72	TMD* n = 46	No TMD n = 26	p-value**
Girls, n (%)	32.0 (44.4)	21.0 (29.2)	11.0 (15.3)	0.784
Age at JIA onset, median (IQR)	7.0 (7.6, 3.0–10.7)	7.5 (7.3, 3.3–10.6)	6.6 (8.5, 2.6–11.1)	0.759
Age at clinical investigation, median (IQR)	13.1 (4.9, 10.2–15.1)	12.9 (4.3, 10.6–14.9)	13.6 (7.6, 7.8–15.4)	0.721
Disease duration, median (IQR)	4.5 (5.5, 2.2–7.7)	4.6 (5.5, 2.2–7.7)	4.1 (5.8, 2.1–8.0)	0.979
**JIA categories, n (%)**				
Oligoarthritis persistent	31.0 (43.7)	19.0 (39.1)	12.0 (52.0)	0.837
Oligoarthritis extended	6.0 (8.5)	3.0 (6.5)	3.0 (12.0)	
Systemic arthritis	1.0 (1.4)	1.0 (2.2)	0.0 (0)	
RF-negative polyarthritis	14.0 (18.3)	9.0 (19.6)	5.0 (16.0)	
Psoriatic arthritis	2 (2.8)	1.0 (2.2)	1.0 (4.0)	
Enthesitis-related arthritis	7.0 (9.9)	6.0 (13.0)	1.0 (4)	
Undifferentiated JIA	11.0 (15.5)	7.0 (17.4)	4.0 (12.0)	

^*^TMD is defined by painful palpation at or around the lateral pole of the TMJ and/or symptoms of painful jaw movements

^**^Chi^2^ -test/Student’s t-test

### Outcome on patient level

**Fig. 2:**
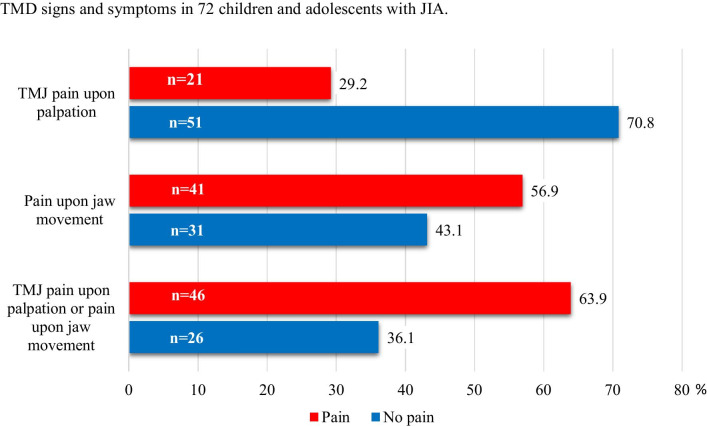
TMD: Temporomandibular disorder; TMJ: Temporomandibular joint; JIA: juvenile idiopathic arthritis

**Fig. 3:**
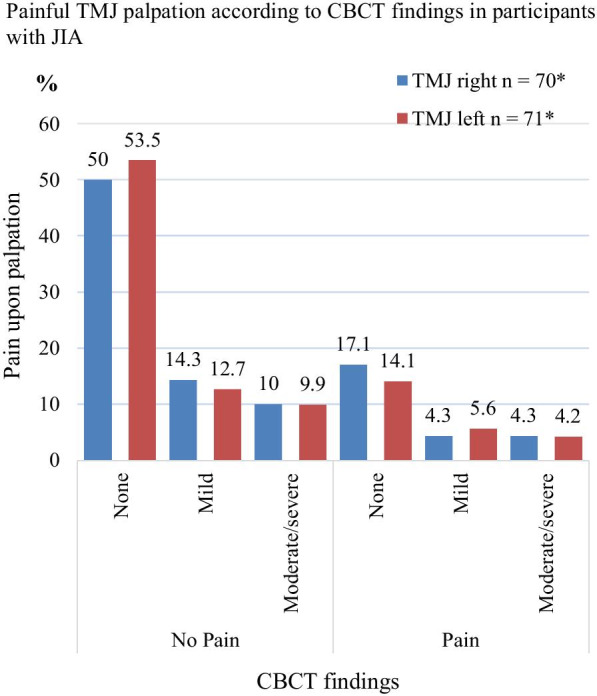
*Three CBCT scans are not available for these analyses because the field of view did not cover the relevant structures. CBCT: Cone-beam computed tomography; TMJ: Temporomandibular joint

**Fig. 4:**
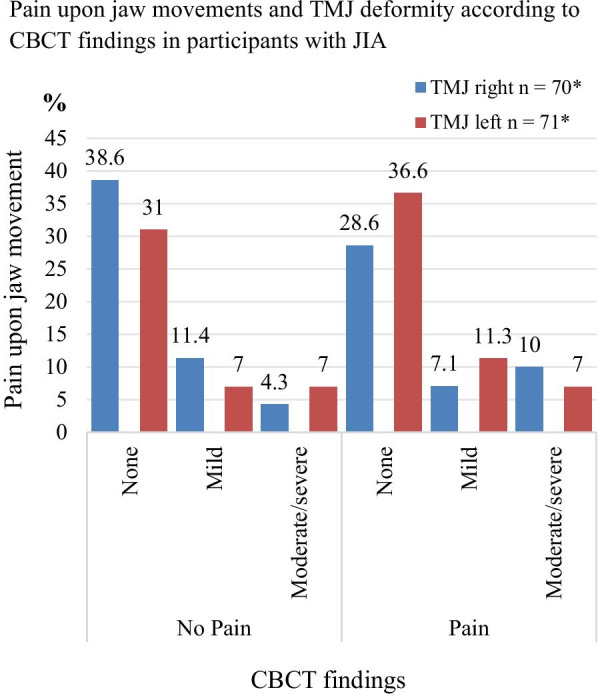
*Three CBCT scans are not available for these analyses because the field of view did not cover the relevant structures

Twenty-one of the 72 participants (29%, 12 girls) experienced pain on palpation at and around the lateral pole, while 41 (56.9%) reported TMJ pain on jaw movement (Fig. 2). Eighteen (25.0%) of the participants were positive to both findings**.** The reason for the inconsequent adding of the numbers are since the mentioned categories overlap and are not mutually exclusive. Mild or moderate/severe TMJ deformity was found in 19 of the 51 (26.4%) participants without pain on palpation at and around the lateral pole, while mild or moderate/severe TMJ deformity was found in 8 of the 21 (11.1%) patients with pain (p = 0.711). TMJ deformity was seen in 10 of the 31 (13.4%) participants without pain on jaw movement but 17 of 41 patients (23.6%) had pain (p = 0.333). No association was seen for either palpatory pain or for pain upon jaw movement between boys and girls (p = 0 0.164, p = 0.588) and between right and left or both TMJ (p = 0.784, p = 0.237). CBCT findings grouped according to pain on palpation and painful jaw movement for right and left side (separately) are presented in detail in Figs. 3 and 4. The distribution of painful palpation of TMJs, painful jaw movements, and structural deformities is presented in Additional file 1: Table S1. Seventeen participants had CBCT findings of deformities in both TMJs, 12 of whom were girls (p = 0.018).

### Outcome on joint level

Of 141 TMJs, 18.4% showed mild and 14.2% showed moderate/severe TMJ deformity visible in CBCT. No statistically significant associations were seen between pain on palpation and TMJ deformity visible in CBCT (p = 0.96 right side and p = 0.38 left side, respectively), or between pain on jaw movement and CBCT findings (p = 0.45 right side and p = 0.84 left side). No association between TMJ deformity and combined pain outcome (pain on both palpation and jaw movement) was seen, with p-values of 0.603 and 0.067 for the right and left TMJ, respectively. Statistical significance was found between CBCT findings and a combined pain outcome (pain at both palpation and jaw movement) in both TMJs for the persistent oligoarticular subtype (p = 0.031).

## Discussion

We have shown that nearly one-third of patients with JIA had pain on palpation at and around the lateral TMJ-pole, and that nearly 60% experienced painful jaw movements. Moreover, assessment by CBCT showed that one-third of the TMJs in these patients was associated with structural deformities, more often in girls than in boys. No associations were seen between pain on palpation of TMJs or painful jaw movements and structural deformities visible with CBCT. The persistent oligoarticular subtype of JIA revealed an association between structural deformities visible with CBCT and clinical signs and symptoms.

The lack of association between clinical signs/symptoms and structural deformity on CBCT in patients with JIA is in line with previous studies that used panoramic radiography as a diagnostic modality [25–27]. In addition, two older studies concluded that asymmetries of mandibular condyles and rami are part of the expected morphological variation in healthy children and adolescents [28, 29], and facial development that might be thought of as disadvantageous may be prevalent among healthy children without a diagnosis of JIA [30]. Only a few CBCT studies have examined structural changes and condylar 3D asymmetry in young individuals with JIA [31–33]. One case–control study of 23 patients with JIA (14 girls, mean age 13.6) using CBCT reported that 83% of the participants had severe structural changes, including cases of extreme deformity even if asymptomatic [31], although the authors did not categorize the extent of JIA.

In this study, we were able to define TMJ deformity in CBCT as either mild or moderate/severe because bony deformities on the condylar surfaces of young individuals are readily detectable using CBCT scans [34, 35]. Other studies have reported differences in terms of condylar flattening [16, 36, 37]. For example, a study of 15 young patients with JIA (mean age 16.3 years old) found signs and symptoms suggestive of TMD in 25 of the 30 TMJs, of which 67% showed condylar flattening based on CBCT scans of 1 mm slice thickness [16]. Similarly, Urtane and colleagues found that 95% of 65 patients with JIA (10–17 years old) had condylar surface flattening based on an even lower slice thickness of 0.3 mm and that there was a correlation (although weakly supported) between pain and condylar surface flattening visible in CBCT imaging [37]. Both studies depicted numerous CBCT scans with distinct anterior condylar flattening but neither of them analysed nor particularly highlighted this flattening. We would argue that this condylar flattening might represent normal variations, as previously shown in several studies [38–40]. In their recent study of panoramic radiographs of 65 children (mean age 12 years old), Cedströmer and colleagues pointed out that even minor bony deformities might hamper craniofacial development [41]. However, our study shows that there is a significant difference between the oligoarticular and polyarticular subtypes. Similar to the results reported by Twilt and colleagues in their panoramic radiograph study of 89 patients (mean age 11.5 years), TMJ deformity was more prevalent when arthritis had an oligoarticular and RF-negative course [42]. Divergent results for condylar deformities that have been generally reported in the literature are probably due to the use of different scoring systems and different patient populations [26, 43, 44]. Previous studies have also shown that TMJ symptoms and signs are not always predictive of TMJ arthritis or TMJ deformity [45, 46]. For example, asymptomatic patients with structural TMJ deformities were reported in a panoramic radiograph study by Billiau and colleagues (26), which included 46 patients (median age at 9.33 years), 28% of whom exhibited condylar deformity without clinical signs or symptoms, which is similar to our study results [26]. However, their study was based on the research diagnostic criteria RDC/TMD [47], which has been validated for ages 18 years and higher, and the young persons in our study were younger than that. A recent MRI study of 50 patients with JIA (9–16 years old) combined clinical variables related to pain and function, and observed TMJ deformity in 9 of 10 patients [48].

In their retrospective CBCT study of 19 JIA and 19 patients with idiopathic condylar resorption (both groups with a mean age of 15.3 years old), Alimanovic and colleagues [49] reported that 55.2% patients of the JIA cohort presented subjective TMJ symptoms, and 42.1% had pain upon TMJ palpation, similar to our results (Figs. 3 and 4). Furthermore, that paper showed that mildly deformed condyles were the most common CBCT finding in both JIA and idiopathic condylar resorption. In their comparative MRI study of 18 JIA patients and 18 patients with anterior disk displacement (ADD) (both groups 11–19 years old), Kellenberger and colleagues (46) reported significantly more TMJ pain upon clinical examination in the ADD-cohort than in JIA. However, deformity in terms of flattening of condylar and temporal bone was more present in the JIA-cohort, and 72% of these had reduced glenoid fossa depth [50]. Those findings corroborate that TMJ arthritis in JIA may often be asymptomatic [51, 52]. Therefore, it is still unknown whether symptoms and signs originating from TMJ arthritis are associated with TMJ deformity.

Our study had some limitations. First, the number of patients with CBCT findings of structural deformities was relatively low. Second, we used a relatively crude CBCT score. The strengths are the meticulous calibration and standardization work performed for both TMD and CBCT assessments. Nonetheless, for this group of patients, longitudinal, prospective studies should be performed to evaluate deformities in pathologies of the TMJ over time.

## Conclusions

There was no association between painful TMD and CBCT imaging features of the TMJ in patients with JIA, but in the subtype of JIA, persistent oligoarticular type, it was found statistical significance between symptoms and signs of TMJ pain and structural CBCT deformities.

## Supplementary Information

**Additional file 1.** Painful and painless TMJs upon jaw movement and palpation according to ILAR categories.

## Data Availability

The datasets generated and analysed in this study are not publicly available because they contain information that could compromise the individual privacy of research participants. The datasets will be made available from the corresponding author on reasonable request.

## Abbreviations

ADD: Articular disk displacement
CBCT: Cone-beam computed tomography
DC/TMD: Diagnostic criteria for temporomandibular disorder
ICR: Idiopathic condylar resorption
ILAR: International league of associations of rheumatology
JIA: Juvenile idiopathic arthritis
MRI: Magnet resonance imaging
RDC/TMD: Research diagnostic criteria for TMD
TMD: Temporomandibular disorder
TMJ: Temporomandibular joint
TMJaw: Temporomandibular Joint Juvenile Arthritis group
