# Supplementary material for: In children and adolescents with temporomandibular disorder assembled with juvenile idiopathic arthritis - no association were found between pain and TMJ deformities using CBCT
Source: BMC Oral Health. 2021 Oct 12;21:518. doi: 10.1186/s12903-021-01870-z (PMC8513178; doi:10.1186/s12903-021-01870-z)
Supplement: Supplementary file 1 — Additional file 1. Painful and painless TMJs upon jaw movement and palpation according to ILAR categories. [file 12903_2021_1870_MOESM1_ESM.docx]

Supplementary material S1.

Painful and painless TMJs upon jaw movement and palpation according to ILAR categories

| n = patients | JIA Category | Painful TMJ Palpation  Left/Right | TMJ deformity  Left/Right | Painful TMJ Movement  Left/Right | TMJ deformity  Left/Right | Painless TMJ  Palpation  Left/ Right | TMJ deformity  Left/ Right | Painless TMJ  Movement  Left/Right | TMJ deformity  Left/Right |
| --- | --- | --- | --- | --- | --- | --- | --- | --- | --- |
| 31 | Oligoarthritis persistent | 8/8 | 4/3 | 16/14 | 6/5 | 23/23 | 8/5 | 15/17 | 6/3 |
| 6 | Oligoarthritis extended | 2/2 | 2/2 | 3/2 | 3/2 | 4/4 | 3/4 | 3/4 | 2/4 |
| 1 | Systemic arthritis | 1/1 | 0/0 | 1/1 | 0/0 | 0/0 | 0/0 | 0/0 | 0/0 |
| 14 | RF-negative polyarthritis | 2/3 | 0/1 | 7/4 | 2/3 | 12/11 | 4/6 | 7/10 | 2/4 |
| 2 | Psoriatic arthritis | 1/1 | 0/0 | 0/0 | 0/0 | 1/1 | 0/0 | 2/2 | 0/0 |
| 7 | Enthesitis-related arthritis | 3/3 | 1/1 | 6/6 | 1/1 | 4/4 | 0/1 | 1/1 | 0/0 |
| 11 | Undifferentiated JIA | 1/2 | 0/1 | 6/6 | 1/1 | 10/9 | 1/1 | 5/5 | 0/0 |
|  | Total TMJ involved left/right | **18/20** | **7/8** | **39/33** | **13/12** | **54/52** | **16/17** | **33/39** | **10/11** |
